# Admission C-reactive protein-to-albumin ratio and serum creatinine in relation to in-hospital mortality in non-HIV tuberculous meningitis: a retrospective cohort study

**DOI:** 10.3389/fmed.2026.1905776

**Published:** 2026-10-05

**Authors:** Wengao Zeng, Xiangzhi Xiao, Qing Liu, Yan Ouyang, Jue Hu, Zhen Wang, Qiong Wu

**Affiliations:** 1 Department of Neurology, The Second Hospital of Changsha (West Branch of Changsha Hospital for Maternal & Child Health Care), Changsha, Hunan, China; 2 Department of Gastroenterology, The Second Hospital of Changsha (West Branch of Changsha Hospital for Maternal & Child Health Care), Changsha, Hunan, China; 3 Department of Neurology, The Affiliated Changsha Central Hospital, Hengyang Medical School, University of South China, Changsha, Hunan, China; 4 Department of Neurosurgery, The Affiliated Changsha Central Hospital, Hengyang Medical School, University of South China, Changsha, Hunan, China

**Keywords:** tuberculous meningitis, C-reactive protein-to-albumin ratio, serum creatinine, in-hospital mortality, nonlinear association, multiple imputation

## Abstract

**Background:**

The independent and joint associations of admission C-reactive protein-to-albumin ratio (CAR) and serum creatinine with mortality in tuberculous meningitis (TBM) remain uncertain.

**Methods:**

We analyzed a single-center retrospective cohort of non-HIV patients hospitalized with TBM from October 2013 to January 2024. Exposure measurements were the first tests within 24 h of admission, before acute interventions. Multivariable logistic regression modeled both exposures continuously, adjusted for age nonlinearly and prespecified covariates, and tested exposure nonlinearity and interaction. Covariate-only multiple imputation generated 20 datasets. Cohort-specific upper-quartile groups were descriptive. An adult sensitivity analysis used estimated glomerular filtration rate (eGFR) calculated with the 2021 CKD-EPI equation.

**Results:**

Among 1,574 eligible patients, 187 died in hospital. The primary analysis included 1,563 patients and all 187 deaths; 11 survivors lacked CAR (*n* = 3) or creatinine (*n* = 8), without overlap. Creatinine showed evidence of nonlinearity (*P* = 0.022), whereas CAR did not (*P* = 0.105). The linear CAR estimate was near the null (adjusted odds ratio [OR] per doubling, 1.02; 95% confidence interval [CI] 0.88–1.18); the interaction *P* value was 0.065. Relative to serum creatinine of 48 µmol/L, adjusted ORs for death were 1.31 (95% CI: 1.11–1.55) at 65 µmol/L, 1.49 (1.15–1.92) at 80 µmol/L, and 1.62 (1.17–2.24) at 100 µmol/L. Descriptive group mortality ranged from 9.02% when both markers were below their 75th percentiles to 19.38% when both were at or above them. Among 1,372 adults, the OR per 10 mL/min/1.73 m^2^ lower eGFR was 1.045 (0.984–1.109; *P* = 0.150).

**Conclusions:**

Admission creatinine had a nonlinear adjusted association with in-hospital mortality in non-HIV TBM. CAR yielded a near-null linear estimate, and the interaction analysis did not establish effect modification. Unmeasured neurological severity limits interpretation; these results do not define kidney dysfunction, a clinical cutoff, or a clinically useful prediction model.

## Introduction

Tuberculous meningitis (TBM) is the most severe form of tuberculosis and remains associated with substantial mortality and neurological disability despite antimicrobial therapy and adjunctive corticosteroids (1–5). The modified British Medical Research Council (BMRC) TBM severity grade classifies patients from grade I to III using the Glasgow Coma Scale (GCS) and focal neurological signs; higher grade is strongly associated with mortality (1, 5, 6). Impaired consciousness, treatment delay, CSF abnormalities, hydrocephalus, and cerebral infarction are also associated with poor outcomes (5–9). These factors remain central to clinical assessment, but routinely available admission laboratory measurements may provide complementary information about systemic illness.

A recent prospective TBM cohort associated higher serum C-reactive protein (CRP) with poor outcome (10). CRP is an acute-phase reactant (11), whereas albumin during acute illness reflects inflammation, hepatic synthesis, capillary leakage, catabolism, and nutritional state rather than nutrition alone (12). CAR combines two nonspecific markers that often change in opposite directions during systemic inflammation and has been associated with mortality in other severe infections (13, 14). Serum creatinine is also widely available, but its concentration reflects glomerular filtration together with age, sex, muscle mass, catabolism, hydration, and assay characteristics (15, 16). A single admission value cannot determine whether a difference is acute or establish chronic kidney disease without temporal and clinical information (17).

CAR and creatinine therefore represent different, partly overlapping dimensions of acute systemic illness. In TBM, however, it remains uncertain whether each is associated with in-hospital mortality after mutual adjustment, whether either association is nonlinear, and whether the creatinine association varies with CAR. These questions are not adequately addressed by cohort-derived categories, which discard information and may not generalize, or by assuming a linear exposure-response relation (18, 19).

We therefore examined the mutually adjusted associations of admission CAR and serum creatinine with all-cause in-hospital mortality in patients with non-HIV TBM. The primary analyses retained both exposures as continuous variables, allowed for nonlinearity, and formally tested CAR-by-creatinine interaction. Cohort-specific upper-quartile groups were used only to describe joint exposure patterns, and an adult sensitivity analysis replaced creatinine with eGFR calculated using the 2021 CKD-EPI creatinine equation (20).

## Methods

### Study design and participants

We conducted a single-center retrospective cohort study using electronic medical records from Changsha Central Hospital. Consecutive patients hospitalized with TBM between October 2013 and January 2024 were screened. Patients with documented HIV co-infection, another intracranial infection, or insufficient clinical information to establish eligibility were excluded. The 123 exclusions for incomplete clinical information lacked CSF and/or biochemical results obtained within 48 h, including one or more of CSF glucose, CSF chloride, CSF white blood cell count, CSF mononuclear-cell percentage, electrolytes, liver function, kidney function, or complete blood count. The specific missing fields and their overlap could not be reconstructed for individual excluded records, so variable-specific exclusion counts were unavailable.

We retained the diagnostic-certainty category recorded in the source dataset: definite, probable, or possible TBM. These categories were based on the clinical record and interpreted with reference to the consensus framework of Marais et al. (21). At the study institution, a definite diagnosis was recorded when supported by CSF acid-fast bacillus staining, Mycobacterium tuberculosis culture, Xpert MTB/RIF, CSF DNA detection by next-generation sequencing, or qualifying histopathologic evidence (22, 23). A sensitivity analysis was restricted to definite or probable TBM.

The Ethics Committee of Changsha Central Hospital approved the study (Approval No. 20250122) and waived written informed consent because the analysis used retrospective anonymized data. Reporting followed the STROBE statement (24). Two earlier reports used the same institutional source cohort to examine drug-induced liver injury and hydrocephalus (25, 26); neither evaluated the present exposures or contributed effect estimates to this analysis.

### Outcome and admission exposures

The outcome was all-cause death during the index hospitalization. CRP (mg/L), albumin (g/L), and serum creatinine (µmol/L) were the first measurements obtained within 24 h of admission and before antituberculosis treatment, corticosteroids, intravenous fluids, or other acute interventions. CAR was calculated as CRP (mg/L) divided by albumin (g/L), yielding the reported numeric ratio in mg/g. Assay-platform, reagent, and calibration records were not consistently available across 2013–2024.

CAR and serum creatinine were analyzed as continuous log2-transformed exposures. For secondary description, higher CAR and higher creatinine denoted values at or above the complete-cohort 75th percentiles (CAR, 0.417; creatinine, 64.75 µmol/L). Group assignment used the unrounded empirical percentiles; the CAR value is reported to three decimal places for presentation. These cohort-derived cut points were used only to construct descriptive groups and were not treated as renal-function or clinical thresholds.

### MRI classification

Two radiologists, blinded to in-hospital outcome, independently reread the first MRI obtained during admission. Each scan was assigned to one of five mutually exclusive categories using a study-specific prespecified hierarchy informed by recognized TBM MRI manifestations: spinal involvement, complicated intracranial lesions, meningeal enhancement, parenchymal lesions, or normal findings (27–29). When more than one pattern was present, the scan was assigned to the highest-ranking applicable category. This hierarchy reduced coexisting imaging patterns to a single category for analysis; it was not treated as a validated MRI severity scale. Disagreements were resolved by discussion with a neurologist. Hydrocephalus, cerebral infarction, and cerebral hemorrhage were additionally recorded as separate present/absent variables. Meningeal enhancement was not recorded as a separate binary feature; it was available only as one level of the mutually exclusive hierarchy. Adjusted models included the mutually exclusive MRI category and did not also include the three separate findings.

### Covariates and missing data

The prespecified primary adjustment set included age, sex, secondary immunodeficiency, hyponatremia, hemoglobin, procalcitonin, TBM diagnostic certainty, and the mutually exclusive MRI category. Age was represented with a three-degree-of-freedom natural spline. An expanded model additionally included CSF protein, CSF glucose, CSF chloride, CSF white blood cell count, and intracranial pressure. The source dataset did not contain retrievable baseline GCS scores, modified BMRC grades, or treatment-delay data; these variables therefore could not be included.

The outcome and exposure variables were not imputed. Multiple imputation by chained equations was restricted to incomplete modeled covariates under a missing-at-random working assumption conditional on observed variables (30, 31). Procalcitonin was the only incomplete modeled covariate, with two missing values among the 1,563 patients in the primary analysis cohort (0.13%). Twenty imputed datasets were used in view of this limited covariate missingness; predictive mean matching was run for 20 iterations. Analyses were fitted within each completed dataset and combined using Rubin's rules. Chain mean and standard-deviation traces were inspected, and a complete-covariate expanded-model sensitivity analysis was performed.

### Statistical analysis

Continuous variables were summarized as medians and interquartile ranges and categorical variables as counts and percentages. Between-outcome comparisons used Wilcoxon rank-sum, chi-square, or Fisher exact tests as descriptive analyses. Primary multivariable logistic models included continuous CAR and creatinine together. Natural splines with three degrees of freedom assessed departures from linearity (19). Nested Rubin-pooled D1 tests assessed the added linear terms, nonlinear terms, and CAR-by-creatinine interaction. Creatinine spline contrasts were expressed relative to the observed cohort median. The prespecified four-category display used the 75th-percentile values only for descriptive mortality and a formal Cochran-Armitage trend test.

Sensitivity analyses used the expanded CSF/intracranial-pressure model, complete covariate records, definite/probable TBM, and CKD-EPI 2021 creatinine-based eGFR among patients aged 18 years or older (20). Collinearity was assessed in every imputed dataset. Covariates were chosen *a priori* on clinical and methodological grounds rather than selected according to observed outcome associations.

Analyses used R version 4.5.3 (R Foundation for Statistical Computing, Vienna, Austria). Two-sided *P* < 0.05 was considered conventional statistical evidence; interaction and nonlinearity tests were interpreted separately from descriptive group trends.

## Results

### Cohort and baseline characteristics

Among 1,737 screened patients, 21 with HIV, 123 with incomplete eligibility information, and 19 with another intracranial infection were excluded, leaving 1,574 eligible patients; 187 died in hospital. Of the eligible patients, 3 lacked CAR because CRP was missing and 8 lacked serum creatinine. These missing-exposure groups did not overlap, all 11 patients were survivors, and in-hospital outcome was complete. The primary exposure-outcome cohort therefore included 1,563 patients and all 187 deaths (Figure 1). The cohort included 1,035 females and 539 males. The selected primary nonlinear model contained 18 predictor degrees of freedom (excluding the intercept) across 187 outcome events. Baseline characteristics are summarized in Table 1; secondary cohort-specific group mortality is shown in Figure 2. Variable-level missingness and full regression coefficients are reported in Supplementary Tables S1, S2, and sensitivity estimates in Supplementary Table S3.

**Figure 1:**
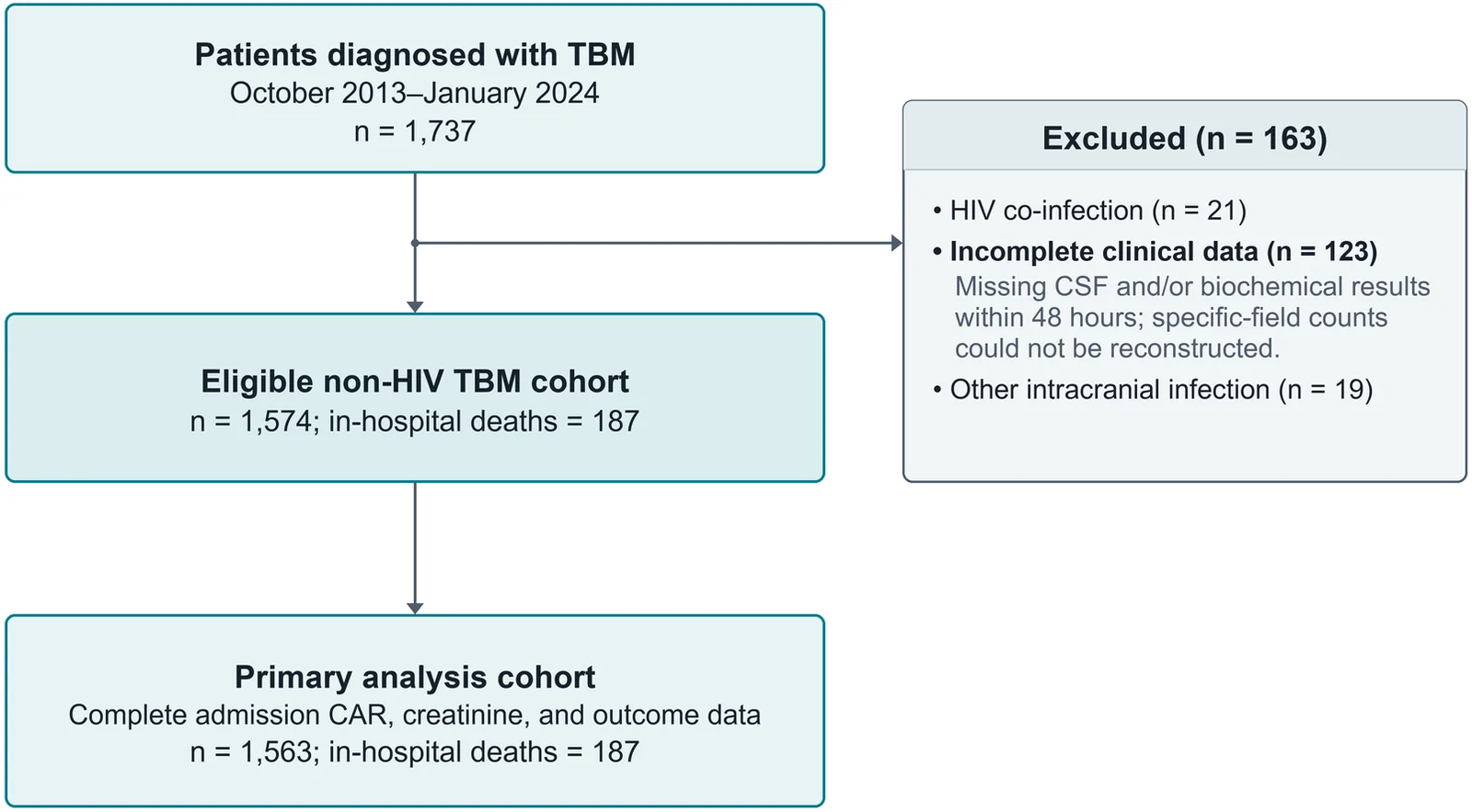
Cohort selection. The 123 incomplete records lacked one or more CSF and/or biochemical results within 48 h; counts attributable to individual missing fields could not be reconstructed. Among the 1,574 eligible patients, 3 lacked CAR because CRP was missing and 8 lacked serum creatinine; the groups did not overlap, all 11 were survivors, and outcome data were complete. The primary analysis therefore included 1,563 patients and all 187 deaths.

**Table 1:** Admission characteristics by in-hospital outcome.

Characteristic	Overall	Survived	Died	*P* value
	*N* = 1,574	*N* = 1,387	*N* = 187	
*N*	1,574	1,387	187	
Age, years	44 [25, 59]	44 [25, 59]	45 [28, 62]	0.055
CRP, mg/L	5.00 [5.00, 14.60]	5.00 [5.00, 14.10]	5.00 [5.00, 15.30]	0.010
Albumin, g/L	35.00 [32.00, 38.50]	35.00 [32.50, 38.80]	34.30 [29.05, 36.95]	<0.001
CAR, mg/g	0.14 [0.13, 0.42]	0.14 [0.12, 0.41]	0.16 [0.14, 0.49]	0.001
Serum creatinine, µmol/L	48.00 [36.00, 65.00]	47.00 [36.00, 63.00]	54.40 [36.10, 77.25]	<0.001
Procalcitonin, ng/mL	0.04 [0.03, 0.09]	0.04 [0.03, 0.08]	0.05 [0.03, 0.28]	<0.001
Hemoglobin, g/L	114.15 [105.00, 126.00]	115.00 [105.00, 126.00]	110.00 [101.85, 119.00]	<0.001
CSF protein, g/L	0.90 [0.59, 1.37]	0.90 [0.58, 1.32]	0.91 [0.60, 1.83]	0.013
CSF chloride, mmol/L	124.20 [115.00, 125.00]	125.00 [116.00, 125.00]	121.00 [113.00, 125.00]	0.016
CSF glucose, mmol/L	2.20 [2.20, 3.21]	2.20 [2.20, 3.21]	2.20 [1.95, 3.20]	0.180
CSF white blood cells, ×10^6^/L	10.00 [10.00, 60.00]	10.00 [10.00, 50.50]	11.00 [10.00, 100.00]	0.031
Intracranial pressure, mmH_2_O	150.00 [120.00, 190.00]	150.00 [125.00, 190.00]	150.00 [120.00, 220.00]	0.209
Female	1,035 (65.8)	906 (65.3)	129 (69)	0.363
Male	539 (34.2)	481 (34.7)	58 (31)	
No secondary immunodeficiency	1,122 (71.3)	1,013 (73)	109 (58.3)	<0.001
Secondary immunodeficiency	452 (28.7)	374 (27)	78 (41.7)	
No hyponatremia	1,132 (71.9)	1,020 (73.5)	112 (59.9)	<0.001
Hyponatremia	442 (28.1)	367 (26.5)	75 (40.1)	
Higher creatinine (≥75th percentile)	393 (25.1)	326 (23.6)	67 (35.8)	<0.001
Lower creatinine (<75th percentile)	1,173 (74.9)	1,053 (76.4)	120 (64.2)	
Definite TBM	553 (35.1)	493 (35.5)	60 (32.1)	0.158
Probable TBM	953 (60.5)	830 (59.8)	123 (65.8)	
Possible TBM	68 (4.3)	64 (4.6)	4 (2.1)	
MRI: normal	226 (14.4)	205 (14.8)	21 (11.2)	0.175
MRI: parenchymal lesions	1,068 (67.9)	939 (67.7)	129 (69)	
MRI: meningeal enhancement	160 (10.2)	140 (10.1)	20 (10.7)	
MRI: complicated intracranial lesions	81 (5.1)	66 (4.8)	15 (8)	
MRI: spinal involvement	39 (2.5)	37 (2.7)	2 (1.1)	

Values are median [IQR] or *n* (%). Denominators vary for variables with missing measurements; missing counts are reported in Supplementary Table S1. *P* values are descriptive and were not used for covariate selection. Female and male counts reflect the confirmed coding: 1 = female and 2 = male. CAR, C-reactive protein-to-albumin ratio; CSF, cerebrospinal fluid; TBM, tuberculous meningitis.

**Figure 2:**
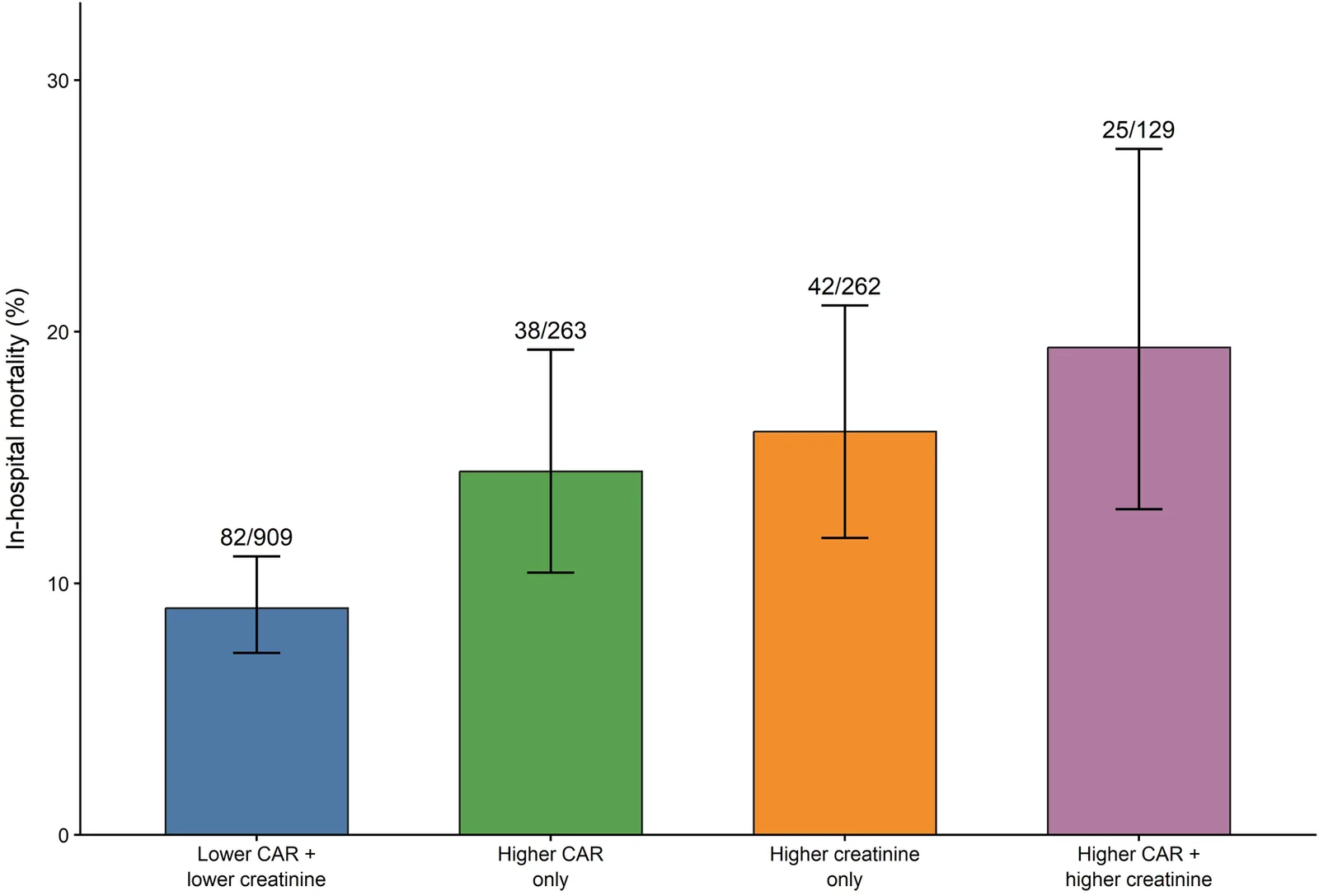
Descriptive in-hospital mortality proportions and exact 95% confidence intervals across cohort-specific CAR-creatinine groups. Labels show deaths/total. Cohort 75th percentiles were used only for description and do not define clinical cutoffs.

### Continuous CAR and creatinine associations

Evidence of nonlinearity was detected for serum creatinine (*P* = 0.022) but not for CAR (*P* = 0.105); jointly adding both nonlinear components improved model fit (*P* = 0.012). In the corresponding linear model, the CAR estimate was close to the null (OR per doubling, 1.02; 95% CI: 0.88–1.18; *P* = 0.806). Using a serum creatinine concentration of 48 µmol/L (the cohort median) as the reference, adjusted ORs were 0.69 at 30 µmol/L, 0.84 at 40 µmol/L, 1.31 at 65 µmol/L, 1.49 at 80 µmol/L, and 1.62 at 100 µmol/L (Table 2). The CAR-by-creatinine interaction test did not meet the conventional evidence threshold (*P* = 0.065). Adjusted continuous curves are shown in Figure 3.

**Table 2:** Primary continuous and nonlinear association results.

Contrast or test	Adjusted OR (95% CI) or method	*P* value
CAR, per doubling (linear model)	1.02 (0.88–1.18)	0.806
Creatinine, per doubling (linear model)	1.15 (0.95–1.39)	0.162
CAR nonlinearity	D1 nested test	0.105
Creatinine nonlinearity	D1 nested test	0.022
Joint CAR and creatinine nonlinearity	D1 nested test	0.012
CAR-by-creatinine interaction	D1 nested test	0.065
Creatinine 30 vs. 48 µmol/L	0.69 (0.53–0.90)	
Creatinine 40 vs. 48 µmol/L	0.84 (0.76–0.93)	
Creatinine 65 vs. 48 µmol/L	1.31 (1.11–1.55)	
Creatinine 80 vs. 48 µmol/L	1.49 (1.15–1.92)	
Creatinine 100 vs. 48 µmol/L	1.62 (1.17–2.24)	

Models adjusted for age (natural spline), sex, secondary immunodeficiency, hyponatremia, hemoglobin, procalcitonin, TBM certainty, and MRI category. Selected creatinine contrasts came from the Rubin-pooled natural-spline model using a serum creatinine concentration of 48 µmol/L as the reference.

**Figure 3:**
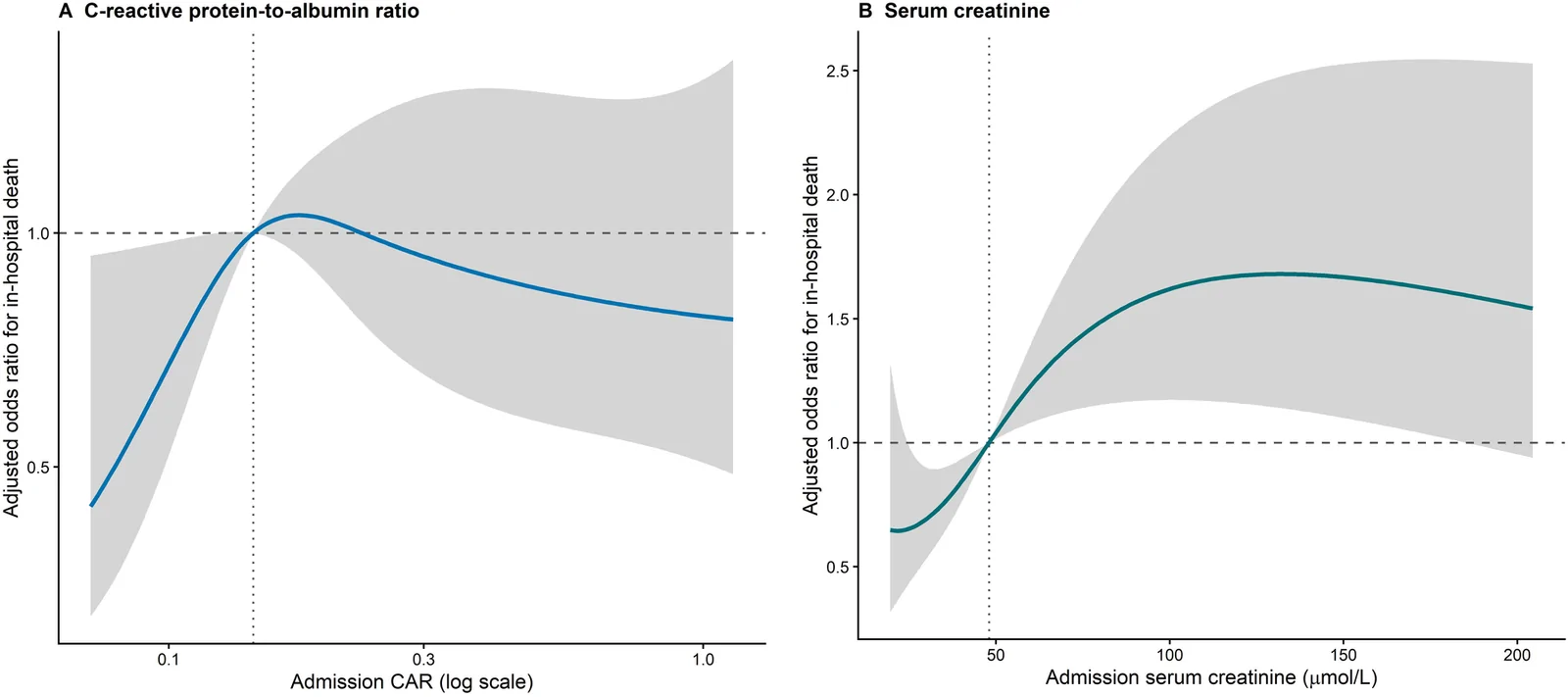
Adjusted continuous associations of admission CAR and serum creatinine with in-hospital death. **Panel A** shows CAR on a logarithmic horizontal scale, with the cohort median CAR (0.143871 mg/g) as reference and creatinine entered linearly. **Panel B** shows the creatinine natural-spline association, using a serum creatinine concentration of 48 µmol/L as the reference and CAR entered linearly. Lines and shaded bands are Rubin-pooled odds ratios and 95% confidence intervals; curves are restricted to the 2.5th−97.5th percentile exposure ranges. Both models adjusted for age nonlinearly, sex, secondary immunodeficiency, hyponatremia, hemoglobin, procalcitonin, TBM certainty, and MRI category.

### Secondary descriptive groups

In the complete exposure-outcome cohort, observed mortality was 9.02%, 14.45%, 16.03%, and 19.38% across the lower-CAR/lower-creatinine, higher-CAR-only, higher-creatinine-only, and higher-CAR/higher-creatinine groups, respectively (Figure 2 and Table 3). A Cochran-Armitage test across this displayed order yielded *P* < 0.001. Because the two middle groups have no inherent clinical ordering and the cut points were cohort-specific, this result was treated as a descriptive pattern rather than evidence of a dose-response relation or synergy.

**Table 3:** Descriptive mortality across cohort-specific CAR-creatinine groups.

Group	*N*	Deaths	Mortality %, 95% CI
Lower CAR + lower creatinine	909	82	9.02 (7.24–11.07)
Higher CAR only	263	38	14.45 (10.43–19.29)
Higher creatinine only	262	42	16.03 (11.80–21.04)
Higher CAR + higher creatinine	129	25	19.38 (12.95–27.26)

### Diagnostic certainty and admission MRI findings

TBM diagnostic certainty differed across the four descriptive CAR-creatinine groups (chi-square = 66.69, 6 degrees of freedom; *P* < 0.001; Supplementary Table S4). The proportion classified as definite TBM decreased from 41.58% in the lower-CAR/lower-creatinine group to 15.50% in the higher-CAR/higher-creatinine group, whereas the proportion classified as possible TBM increased from 2.64% to 11.63%.

On the first admission MRI, hydrocephalus was recorded in 119 of 1,574 patients (7.6%) and cerebral hemorrhage in 16 of 1,574 (1.0%). Infarction status was available for 1,573 patients (one missing record), of whom 265 (16.8%) had cerebral infarction. Hydrocephalus and hemorrhage were more frequent among patients who died, whereas infarction frequency did not differ significantly between survivors and deaths (*P* = 0.253; Supplementary Table S5). In the mutually exclusive hierarchy, 160 scans (10.2%) were assigned to the meningeal-enhancement category; this figure is not the prevalence of enhancement as an independently assessed binary feature.

### Sensitivity analyses

Procalcitonin was missing in 3 of 1,574 eligible patients (0.19%), including 2 of the 1,563 patients in the primary analysis cohort (0.13%); it was the only modeled covariate requiring imputation. Chain diagnostics are shown in Supplementary Figure S1. The expanded CSF/intracranial-pressure model and complete-covariate analysis (*N* = 1,561; 187 deaths) gave similar linear CAR and creatinine estimates. Restricting the analysis to definite or probable TBM included 1,495 patients and 183 deaths and produced estimates close to the primary linear results: the adjusted OR per doubling was 1.03 (95% CI: 0.88–1.20; *P* = 0.708) for CAR and 1.20 (0.99–1.46; *P* = 0.061) for creatinine. Both confidence intervals included 1. This sensitivity analysis addressed diagnostic certainty but did not re-estimate the nonlinear curves or remove confounding by unmeasured disease severity (Supplementary Table S3). Among 1,372 adults with 168 deaths, each 10 mL/min/1.73 m^2^ lower eGFR had an adjusted OR of 1.045 (0.984–1.109; *P* = 0.150). Maximum variance inflation factors remained below 2 across imputed datasets. Laboratory metadata, nested-model tests, and collinearity summaries are provided in Supplementary Tables S6–S8.

## Discussion

In this large retrospective cohort of patients with non-HIV TBM, admission serum creatinine had a nonlinear adjusted association with in-hospital mortality. CAR produced a near-null linear estimate and no detected departure from linearity, while the CAR-by-creatinine interaction test did not meet the conventional evidence threshold. Mortality increased across the four cohort-specific groups, but that descriptive ordering neither established biological synergy nor validated the 75th-percentile values as clinical thresholds. The principal inference therefore rests on the continuous creatinine curve rather than the categorized groups.

These laboratory results must be interpreted in the context of established TBM severity. The modified BMRC grade combines consciousness level, expressed by GCS, with focal neurological signs and has a strong relationship with mortality (1, 5, 6). Our dataset lacked GCS, modified BMRC grade, and treatment-delay data, so the models could not fully account for neurological severity. Adjustment for MRI category and diagnostic certainty does not replace those measures, and residual confounding could explain part of the creatinine association. Hydrocephalus was more frequent among patients who died, but the MRI feature comparisons were unadjusted and do not establish independent effects.

The creatinine curve was characterized by lower adjusted odds at 30 and 40 µmol/L and higher adjusted odds at 65, 80, and 100 µmol/L, each compared with 48 µmol/L. Serum creatinine reflects glomerular filtration but is also influenced by age, sex, muscle mass, catabolism, nutrition, hydration, and assay factors (15, 16). A single admission value therefore cannot distinguish reduced filtration from nonrenal influences, determine whether a change is acute, or establish chronic kidney disease (17). In a large general-hospital cohort, both very low and high admission creatinine were associated with mortality, producing a U-shaped relation (32). The low end of that relation differs from the pattern observed here, underscoring that the shape is population-dependent and should not be assigned a universal kidney-specific mechanism.

The adult eGFR sensitivity analysis also requires careful interpretation. Each 10 mL/min/1.73 m^2^ lower eGFR had an adjusted OR of 1.045, but the 95% CI (0.984–1.109) included 1. Because CKD-EPI eGFR is calculated from creatinine together with age and sex (20), it is not an independent kidney biomarker. Moreover, the eGFR analysis assumed a linear effect per 10-unit decrease and therefore did not directly test the nonlinear shape seen for creatinine. It neither confirms nor refutes a kidney-function-specific explanation.

CAR likewise showed no clear independent signal in this cohort. The OR per doubling was 1.02 (95% CI: 0.88–1.18; *P* = 0.806), a range compatible with either a modest decrease or a modest increase in the odds of death, and no departure from linearity was detected. CRP and albumin are both nonspecific during acute illness (11, 12). The recent TBM CRP study evaluated CRP rather than CAR, enrolled a population in which 83% had HIV, and used severe disability or death at 8 weeks as its outcome (10); the sepsis CAR studies involved different diseases (13, 14). A larger prospective TBM cohort also showed that relationships between mortality and inflammatory features varied across blood and CSF measures and by HIV status (33). These studies are therefore informative context, not direct comparators, and the present result should not be read as evidence that inflammation is unimportant in TBM.

The four-group display remains useful for communicating the observed joint distribution. Mortality was lowest when both markers were below their 75th percentiles (9.02%) and highest when both were at or above them (19.38%). Nevertheless, the cut points were derived from this cohort, dichotomization loses information (18), and the formal continuous interaction test was inconclusive (*P* = 0.065). The groups should therefore be viewed as a descriptive summary, not as a new phenotype, a dose-response scale, or evidence of synergy.

Clinically, these findings do not support a creatinine cutoff, a stand-alone CAR rule, or a change in antituberculosis treatment. A higher admission creatinine value instead warrants interpretation in its clinical context, including hydration, medication exposure, urine output, prior kidney history, and serial measurements. These are general principles for interpreting serum creatinine (15, 17), not interventions tested by this study. CAR should likewise be considered alongside, and not in place of, GCS, modified BMRC grade, CSF findings, and neuroimaging.

The study has several strengths. It included a large consecutive cohort and used the first pre-intervention CRP, albumin, and creatinine measurements. Both exposures were modeled simultaneously as continuous variables, with explicit tests of nonlinearity and interaction; adjustment variables were chosen *a priori* rather than selected from observed outcome associations. Multiple imputation was confined to covariates, and sensitivity analyses addressed CSF and intracranial-pressure measures, complete covariate records, diagnostic certainty, and adult eGFR. Two outcome-blinded radiologists independently reread the first admission MRI using a prespecified classification process.

Several limitations remain. The retrospective single-center design limits external generalizability and permits residual confounding, particularly because GCS, modified BMRC grade, and treatment delay were unavailable. Exclusion of 123 patients with incomplete eligibility information could have introduced selection bias, and variable-specific exclusion counts could not be reconstructed. Assay-platform, reagent, and calibration histories across 2013–2024 were unavailable. A single creatinine measurement, without serial creatinine, urine output, baseline kidney history, cystatin C, or standardized adjudication of acute kidney injury and chronic kidney disease, cannot characterize the nature or duration of kidney abnormalities. Although MRI review was independent and outcome-blinded, formal agreement statistics were not retained. Meningeal enhancement was available only as a hierarchy category, and the findings should not be extrapolated to HIV-associated TBM without direct study.

Future multicenter studies should prospectively record admission GCS, focal neurological findings, modified BMRC grade, and treatment timing; standardize laboratory sampling and assay procedures; and obtain serial creatinine, urine-output, and prior kidney data. Imaging studies should retain reader-level assessments so that agreement can be quantified. External replication should analyze continuous exposure functions and prespecified interactions rather than transplant this cohort's quartile cut points. Such studies are needed to determine whether the creatinine association persists after adequate adjustment for accepted TBM severity measures and whether CAR has a reproducible association in clinically distinct populations.

## Conclusion

Admission serum creatinine was nonlinearly associated with in-hospital mortality in this non-HIV TBM cohort. Using a serum creatinine concentration of 48 µmol/L as the reference, adjusted ORs for death were 1.31 (95% CI: 1.11–1.55) at 65 µmol/L, 1.49 (1.15–1.92) at 80 µmol/L, and 1.62 (1.17–2.24) at 100 µmol/L. CAR yielded a near-null linear estimate, with no detected departure from linearity, and the interaction analysis did not establish that CAR modified the creatinine association. These observational results neither define kidney dysfunction or a clinical cutoff nor establish a clinically useful prediction model; external validation with standardized neurological and kidney characterization is required.

## Data Availability

Code and de-identified data are available from the corresponding author upon reasonable request.
