# Supplementary material for: Admission C-reactive protein-to-albumin ratio and serum creatinine in relation to in-hospital mortality in non-HIV tuberculous meningitis: a retrospective cohort study
Source: Front Med (Lausanne). 2026 Oct 5;13:1905776. doi: 10.3389/fmed.2026.1905776 (PMC13677553; doi:10.3389/fmed.2026.1905776)
Supplement: Supplementary file 1 [file Supplementaryfile1.docx]

# **Supplementary material**

## **Supplementary Table S1. Missingness in analysis variables**

| **Variable** | **Overall missing n (%)** | **Survived missing n (%)** | **Died missing n (%)** |
| --- | --- | --- | --- |
| Age | 0 (0.00) | 0 (0.00) | 0 (0.00) |
| sex | 0 (0.00) | 0 (0.00) | 0 (0.00) |
| Immunodeficiency | 0 (0.00) | 0 (0.00) | 0 (0.00) |
| Hyponatremia | 0 (0.00) | 0 (0.00) | 0 (0.00) |
| Hemoglobin | 0 (0.00) | 0 (0.00) | 0 (0.00) |
| PCT | 3 (0.19) | 3 (0.22) | 0 (0.00) |
| Tuberculous_Meningitis | 0 (0.00) | 0 (0.00) | 0 (0.00) |
| MRI | 0 (0.00) | 0 (0.00) | 0 (0.00) |
| CRP | 3 (0.19) | 3 (0.22) | 0 (0.00) |
| ALB | 1 (0.06) | 1 (0.07) | 0 (0.00) |
| Cr | 8 (0.51) | 8 (0.58) | 0 (0.00) |
| CSF_Protein | 0 (0.00) | 0 (0.00) | 0 (0.00) |
| CSF_Glucose | 0 (0.00) | 0 (0.00) | 0 (0.00) |
| CSF_Chloride | 0 (0.00) | 0 (0.00) | 0 (0.00) |
| CSFWBC | 0 (0.00) | 0 (0.00) | 0 (0.00) |
| ICP | 0 (0.00) | 0 (0.00) | 0 (0.00) |
| GCS | 1574 (100.00) | 1387 (100.00) | 187 (100.00) |
| BMRC | 1574 (100.00) | 1387 (100.00) | 187 (100.00) |
| Treatment_Delay | 1574 (100.00) | 1387 (100.00) | 187 (100.00) |
| Outcome_Death | 0 (0.00) | 0 (0.00) | 0 (0.00) |

Missing counts refer to the full eligible cohort (N=1,574), in which three procalcitonin values were missing. In the primary exposure-outcome cohort (N=1,563), two procalcitonin values were missing and imputed; the complete-covariate analysis included 1,561 patients. Only procalcitonin was imputed among modeled covariates. Exposures and outcome were not imputed. GCS, Glasgow Coma Scale; BMRC, British Medical Research Council stage.

## **Supplementary Table S2. Complete adjusted regression outputs**

Complete adjusted regression outputs are shown for the base covariate model, the base model plus creatinine, the expanded CSF/intracranial-pressure model, and the expanded model plus CAR and creatinine.

**Base covariate model**

| **Term** | **OR** | **95% CI** | **P value** | **N** | **Deaths** |
| --- | --- | --- | --- | --- | --- |
| Intercept | 0.061 | 0.026-0.143 | <0.001 | 1563 | 187 |
| Age natural spline component 1 | 0.764 | 0.383-1.524 | 0.445 | 1563 | 187 |
| Age natural spline component 2 | 1.901 | 0.390-9.258 | 0.426 | 1563 | 187 |
| Age natural spline component 3 | 1.237 | 0.627-2.442 | 0.540 | 1563 | 187 |
| Male sex (vs female) | 0.824 | 0.582-1.166 | 0.273 | 1563 | 187 |
| Secondary immunodeficiency (vs no) | 1.676 | 1.173-2.396 | 0.005 | 1563 | 187 |
| Hyponatremia (vs no) | 1.665 | 1.199-2.313 | 0.002 | 1563 | 187 |
| Hemoglobin (per SD) | 0.842 | 0.724-0.979 | 0.025 | 1563 | 187 |
| Log procalcitonin (per SD) | 1.315 | 1.166-1.484 | <0.001 | 1563 | 187 |
| Probable TBM (vs definite) | 1.034 | 0.729-1.465 | 0.852 | 1563 | 187 |
| Possible TBM (vs definite) | 0.276 | 0.088-0.866 | 0.027 | 1563 | 187 |
| MRI parenchymal lesions (vs normal) | 1.384 | 0.836-2.289 | 0.206 | 1563 | 187 |
| MRI meningeal enhancement (vs normal) | 1.694 | 0.859-3.339 | 0.128 | 1563 | 187 |
| MRI complicated intracranial lesions (vs normal) | 1.990 | 0.939-4.215 | 0.072 | 1563 | 187 |
| MRI spinal involvement (vs normal) | 0.650 | 0.143-2.965 | 0.578 | 1563 | 187 |

**Base covariate model plus continuous creatinine**

| **Term** | **OR** | **95% CI** | **P value** | **N** | **Deaths** |
| --- | --- | --- | --- | --- | --- |
| Intercept | 0.032 | 0.009-0.111 | <0.001 | 1563 | 187 |
| Age natural spline component 1 | 0.698 | 0.345-1.412 | 0.317 | 1563 | 187 |
| Age natural spline component 2 | 1.426 | 0.277-7.352 | 0.671 | 1563 | 187 |
| Age natural spline component 3 | 1.145 | 0.574-2.285 | 0.700 | 1563 | 187 |
| Male sex (vs female) | 0.853 | 0.600-1.211 | 0.373 | 1563 | 187 |
| Secondary immunodeficiency (vs no) | 1.628 | 1.135-2.336 | 0.008 | 1563 | 187 |
| Hyponatremia (vs no) | 1.676 | 1.206-2.330 | 0.002 | 1563 | 187 |
| Hemoglobin (per SD) | 0.853 | 0.733-0.994 | 0.041 | 1563 | 187 |
| Log procalcitonin (per SD) | 1.291 | 1.141-1.461 | <0.001 | 1563 | 187 |
| Probable TBM (vs definite) | 1.015 | 0.715-1.440 | 0.935 | 1563 | 187 |
| Possible TBM (vs definite) | 0.253 | 0.078-0.817 | 0.022 | 1563 | 187 |
| MRI parenchymal lesions (vs normal) | 1.389 | 0.840-2.299 | 0.200 | 1563 | 187 |
| MRI meningeal enhancement (vs normal) | 1.692 | 0.858-3.338 | 0.129 | 1563 | 187 |
| MRI complicated intracranial lesions (vs normal) | 2.006 | 0.947-4.249 | 0.069 | 1563 | 187 |
| MRI spinal involvement (vs normal) | 0.665 | 0.146-3.033 | 0.598 | 1563 | 187 |
| Serum creatinine (per doubling) | 1.145 | 0.946-1.387 | 0.164 | 1563 | 187 |

**Expanded CSF/intracranial-pressure covariate model**

| **Term** | **OR** | **95% CI** | **P value** | **N** | **Deaths** |
| --- | --- | --- | --- | --- | --- |
| Intercept | 0.060 | 0.025-0.143 | <0.001 | 1563 | 187 |
| Age natural spline component 1 | 0.770 | 0.384-1.545 | 0.462 | 1563 | 187 |
| Age natural spline component 2 | 1.895 | 0.384-9.351 | 0.433 | 1563 | 187 |
| Age natural spline component 3 | 1.247 | 0.626-2.481 | 0.530 | 1563 | 187 |
| Male sex (vs female) | 0.818 | 0.577-1.160 | 0.259 | 1563 | 187 |
| Secondary immunodeficiency (vs no) | 1.682 | 1.172-2.414 | 0.005 | 1563 | 187 |
| Hyponatremia (vs no) | 1.618 | 1.108-2.362 | 0.013 | 1563 | 187 |
| Hemoglobin (per SD) | 0.831 | 0.712-0.968 | 0.018 | 1563 | 187 |
| Log procalcitonin (per SD) | 1.309 | 1.159-1.478 | <0.001 | 1563 | 187 |
| Probable TBM (vs definite) | 1.070 | 0.752-1.521 | 0.707 | 1563 | 187 |
| Possible TBM (vs definite) | 0.304 | 0.097-0.952 | 0.041 | 1563 | 187 |
| MRI parenchymal lesions (vs normal) | 1.362 | 0.821-2.261 | 0.232 | 1563 | 187 |
| MRI meningeal enhancement (vs normal) | 1.636 | 0.827-3.234 | 0.157 | 1563 | 187 |
| MRI complicated intracranial lesions (vs normal) | 1.827 | 0.857-3.894 | 0.119 | 1563 | 187 |
| MRI spinal involvement (vs normal) | 0.632 | 0.138-2.890 | 0.554 | 1563 | 187 |
| Log CSF protein (per SD) | 1.136 | 0.939-1.373 | 0.189 | 1563 | 187 |
| CSF glucose (per SD) | 0.935 | 0.794-1.102 | 0.424 | 1563 | 187 |
| CSF chloride (per SD) | 1.127 | 0.919-1.381 | 0.250 | 1563 | 187 |
| Log CSF white blood cell count (per SD) | 1.097 | 0.904-1.331 | 0.347 | 1563 | 187 |
| Log intracranial pressure (per SD) | 1.018 | 0.863-1.201 | 0.831 | 1563 | 187 |

**Expanded model plus continuous CAR and creatinine**

| **Term** | **OR** | **95% CI** | **P value** | **N** | **Deaths** |
| --- | --- | --- | --- | --- | --- |
| Intercept | 0.068 | 0.028-0.165 | <0.001 | 1563 | 187 |
| Age natural spline component 1 | 0.696 | 0.342-1.418 | 0.318 | 1563 | 187 |
| Age natural spline component 2 | 1.354 | 0.255-7.184 | 0.722 | 1563 | 187 |
| Age natural spline component 3 | 1.136 | 0.562-2.294 | 0.723 | 1563 | 187 |
| Male sex (vs female) | 0.852 | 0.598-1.213 | 0.374 | 1563 | 187 |
| Secondary immunodeficiency (vs no) | 1.625 | 1.127-2.343 | 0.009 | 1563 | 187 |
| Hyponatremia (vs no) | 1.599 | 1.092-2.342 | 0.016 | 1563 | 187 |
| Hemoglobin (per SD) | 0.848 | 0.722-0.996 | 0.044 | 1563 | 187 |
| Log procalcitonin (per SD) | 1.278 | 1.123-1.454 | <0.001 | 1563 | 187 |
| Probable TBM (vs definite) | 1.050 | 0.737-1.496 | 0.786 | 1563 | 187 |
| Possible TBM (vs definite) | 0.279 | 0.087-0.894 | 0.032 | 1563 | 187 |
| MRI parenchymal lesions (vs normal) | 1.365 | 0.821-2.268 | 0.230 | 1563 | 187 |
| MRI meningeal enhancement (vs normal) | 1.638 | 0.828-3.242 | 0.156 | 1563 | 187 |
| MRI complicated intracranial lesions (vs normal) | 1.841 | 0.864-3.924 | 0.114 | 1563 | 187 |
| MRI spinal involvement (vs normal) | 0.641 | 0.140-2.934 | 0.567 | 1563 | 187 |
| Log CSF protein (per SD) | 1.139 | 0.942-1.378 | 0.178 | 1563 | 187 |
| CSF glucose (per SD) | 0.930 | 0.789-1.096 | 0.388 | 1563 | 187 |
| CSF chloride (per SD) | 1.116 | 0.910-1.369 | 0.290 | 1563 | 187 |
| Log CSF white blood cell count (per SD) | 1.093 | 0.901-1.327 | 0.367 | 1563 | 187 |
| Log intracranial pressure (per SD) | 1.017 | 0.862-1.200 | 0.840 | 1563 | 187 |
| CAR (per doubling) | 1.024 | 0.881-1.191 | 0.754 | 1563 | 187 |
| Serum creatinine (per doubling) | 1.153 | 0.951-1.397 | 0.148 | 1563 | 187 |

## **Supplementary Table S3. Sensitivity analyses**

| **Analysis** | **Exposure** | **Adjusted OR (95% CI)** | **P value** | **N** | **Deaths** |
| --- | --- | --- | --- | --- | --- |
| Complete-covariate expanded model | CAR per doubling | 1.03 (0.88-1.20) | 0.689 | 1,561 | 187 |
| Complete-covariate expanded model | Creatinine per doubling | 1.15 (0.94-1.39) | 0.160 | 1,561 | 187 |
| Definite/probable TBM | CAR per doubling | 1.03 (0.88-1.20) | 0.708 | 1,495 | 183 |
| Definite/probable TBM | Creatinine per doubling | 1.20 (0.99-1.46) | 0.061 | 1,495 | 183 |
| Adults aged ≥18 years | eGFR per 10 mL/min/1.73 m² lower | 1.04 (0.98-1.11) | 0.150 | 1,372 | 168 |

## **Supplementary Table S4. TBM diagnostic certainty across descriptive CAR-creatinine groups**

| **CAR-creatinine group** | **Diagnostic certainty** | **N** | **Group total** | **Within-group %** |
| --- | --- | --- | --- | --- |
| Lower CAR + lower creatinine | Definite | 378 | 909 | 41.58 |
| Lower CAR + lower creatinine | Probable | 507 | 909 | 55.78 |
| Lower CAR + lower creatinine | Possible | 24 | 909 | 2.64 |
| Higher CAR only | Definite | 79 | 263 | 30.04 |
| Higher CAR only | Probable | 167 | 263 | 63.50 |
| Higher CAR only | Possible | 17 | 263 | 6.46 |
| Higher creatinine only | Definite | 70 | 262 | 26.72 |
| Higher creatinine only | Probable | 180 | 262 | 68.70 |
| Higher creatinine only | Possible | 12 | 262 | 4.58 |
| Higher CAR + higher creatinine | Definite | 20 | 129 | 15.50 |
| Higher CAR + higher creatinine | Probable | 94 | 129 | 72.87 |
| Higher CAR + higher creatinine | Possible | 15 | 129 | 11.63 |

*Pearson chi-square=66.69, df=6, P<0.001; minimum expected cell count=5.61. This is a descriptive composition test.*

## **Supplementary Table S5. First admission MRI findings by in-hospital outcome**

| **MRI finding** | **Evaluated N** | **Overall** | **Survived** | **Died** | **P value** |
| --- | --- | --- | --- | --- | --- |
| Hydrocephalus | 1574 | 119 (7.6%) | 88 (6.3%) | 31 (16.6%) | <0.001 |
| Cerebral infarction | 1573 | 265 (16.8%) | 228 (16.5%) | 37 (19.8%) | 0.253 |
| Cerebral hemorrhage | 1574 | 16 (1.0%) | 11 (0.8%) | 5 (2.7%) | 0.033 |
| Meningeal enhancement (hierarchy category) | 1574 | 160 (10.2%) | 140 (10.1%) | 20 (10.7%) | 0.797 |

*Values are positive n (%). P values are descriptive Fisher exact tests. Hydrocephalus, cerebral infarction, and cerebral hemorrhage were separate binary fields. Meningeal enhancement was retained only as a level of the mutually exclusive MRI hierarchy and was not available as a separate binary indicator.*

## **Supplementary Table S6. Admission exposure measurement metadata**

| **Variable** | **Unit** | **Sampling** | **Intervention timing** | **Assay history** |
| --- | --- | --- | --- | --- |
| CRP | mg/L | First measurement within 24 hours of admission | Before antituberculosis treatment, corticosteroids, intravenous fluids, or other acute interventions | Unavailable for the 2013-2024 period; treated as measurement heterogeneity |
| Albumin | g/L | First measurement within 24 hours of admission | Before antituberculosis treatment, corticosteroids, intravenous fluids, or other acute interventions | Unavailable for the 2013-2024 period; treated as measurement heterogeneity |
| Serum creatinine | µmol/L | First measurement within 24 hours of admission | Before antituberculosis treatment, corticosteroids, intravenous fluids, or other acute interventions | Unavailable for the 2013-2024 period; treated as measurement heterogeneity |

## **Supplementary Table S7. Nested association-model tests**

| **Comparison** | **F statistic** | **df1** | **P value** |
| --- | --- | --- | --- |
| Base covariates vs base covariates plus continuous CAR | 0.040 | 1 | 0.842 |
| Base covariates vs base covariates plus continuous creatinine | 1.937 | 1 | 0.164 |
| Base covariates vs base covariates plus continuous CAR and creatinine | 1.000 | 2 | 0.368 |
| Add CAR-by-creatinine interaction to the base model | 3.419 | 1 | 0.065 |
| Expanded covariates vs expanded covariates plus continuous CAR and creatinine | 1.083 | 2 | 0.339 |
| Add CAR-by-creatinine interaction to the expanded model | 3.241 | 1 | 0.072 |
| Add the four descriptive CAR-creatinine groups to the base model | 2.059 | 3 | 0.104 |
| Nonlinearity test: CAR spline vs linear | 2.261 | 2 | 0.105 |
| Nonlinearity test: creatinine spline vs linear | 3.805 | 2 | 0.022 |
| Joint nonlinearity test: CAR and creatinine splines | 3.232 | 4 | 0.012 |

## **Supplementary Table S8. Collinearity summary**

| **Model** | **Term with maximum VIF** | **Maximum VIF** | **Imputations** |
| --- | --- | --- | --- |
| Base covariates plus continuous CAR and creatinine | MRI parenchymal lesions (vs normal) | 1.87 | 20 |
| Base covariates plus CAR-creatinine interaction | Serum creatinine (per doubling) | 1.94 | 20 |
| Expanded covariates plus continuous CAR and creatinine | MRI parenchymal lesions (vs normal) | 1.89 | 20 |
| Expanded covariates plus CAR-creatinine interaction | Serum creatinine (per doubling) | 1.94 | 20 |
| Base covariates plus four descriptive groups | MRI parenchymal lesions (vs normal) | 1.87 | 20 |
| Expanded covariates plus four descriptive groups | MRI parenchymal lesions (vs normal) | 1.89 | 20 |

## **Supplementary Figure S1. Multiple-imputation convergence traces**

Supplementary Figure S1. Chain means and standard deviations across 20 iterations for procalcitonin, the only modeled covariate that was imputed. Visual inspection did not indicate systematic chain drift.
